# Catheter Misplacement Revealing Partial Anomalous Pulmonary Venous Return (PAPVR) in an Acute Kidney Injury Patient

**DOI:** 10.7759/cureus.80830

**Published:** 2025-03-19

**Authors:** Ramy Zughul, Ayham Asassfeh, Rajit Pahwa

**Affiliations:** 1 Critical Care Medicine, University of Michigan, Lansing, USA; 2 Medicine, School of Medicine, University of Jordan, Amman, JOR

**Keywords:** anomalies of vascular anatomy, intensive and critical care, partial anomalous pulmonary venous return (papvr), procedural complications, vascular access procedures

## Abstract

An 84-year-old patient, treated in the ICU for acute kidney injury complicating coronary artery bypass surgery, experienced an unusual event during dialysis catheter placement. First, venous blood was drawn while the catheter was inserted into the left internal jugular vein. Ultrasound confirmed the proper guidewire placement, and the dilator and catheter were inserted without difficulty. However, the blood returned from the catheter was unexpectedly bright, raising concerns for arterial placement. Transduced pressures were pulsatile, but levels were inconsistent with arterial positioning. A subsequent chest x-ray revealed the catheter extending into the left mediastinum, and CT imaging confirmed the catheter was inadvertently placed in an anomalous left pulmonary vein. Misplacement of central venous catheters (CVCs) into partial anomalous pulmonary venous return (PAPVR) has been sporadically reported. As imaging modalities, such as CT and MRI, become more prevalent in intensive care practice, incidental detection of PAPVR is expected to increase. Management typically involves removal and repositioning of the catheter, though the decision to treat the PAPVR itself remains nuanced and is based on factors such as pulmonary hypertension risk and shunt volume.

## Introduction

Central venous catheter (CVC) misplacement into an anomalous pulmonary vein is a rare but important clinical occurrence. CVC malpositioning is generally well-documented, with complications arising in approximately 6.7% of catheter placements, predominantly in left-sided approaches such as the internal jugular or subclavian veins [1]. A CVC is a flexible tube inserted into a large vein to deliver medications, fluids, or dialysis treatment, but improper placement can lead to serious complications.

Partial anomalous pulmonary venous return (PAPVR), a congenital heart defect with an incidence of 0.1%-0.4%, complicates CVC placement as the anomalous pulmonary vein may inadvertently be cannulated [2-5]. PAPVR occurs when one or more pulmonary veins that normally carry oxygenated blood from the lungs to the heart’s left atrium, instead drain into the wrong chamber (such as the right atrium) or a systemic vein. This abnormal blood flow, known as shunt flow, can increase strain on the heart and, if significant, contribute to pulmonary hypertension and heart failure over time [4,5]. While PAPVR is often asymptomatic and typically detected incidentally during imaging, its presence may lead to procedural complications during CVC placement, posing additional challenges in critically ill patients [6].

## Case presentation

This 85-year-old male with a history of coronary artery disease and multiple comorbidities was admitted following a non-ST elevation myocardial infarction (NSTEMI) and underwent coronary artery bypass grafting (CABG) with a left internal mammary artery (LIMA) to the left anterior descending artery (LAD), a modified Maze procedure, and left atrial appendage ligation. His postoperative course was complicated by coagulopathy, respiratory failure requiring high-flow oxygen and bilevel positive airway pressure (BIPAP), worsening acute kidney injury (AKI) on chronic kidney disease (CKD), and atrial fibrillation with rapid ventricular response necessitating cardioversions and amiodarone therapy. Nephrology followed his AKI closely, with continuous renal replacement therapy (CRRT) considered but not initiated. Cardiovascular support was provided with dopamine and later epinephrine for blood pressure management, alongside aggressive diuresis to optimize renal function. 

**Figure 1:**
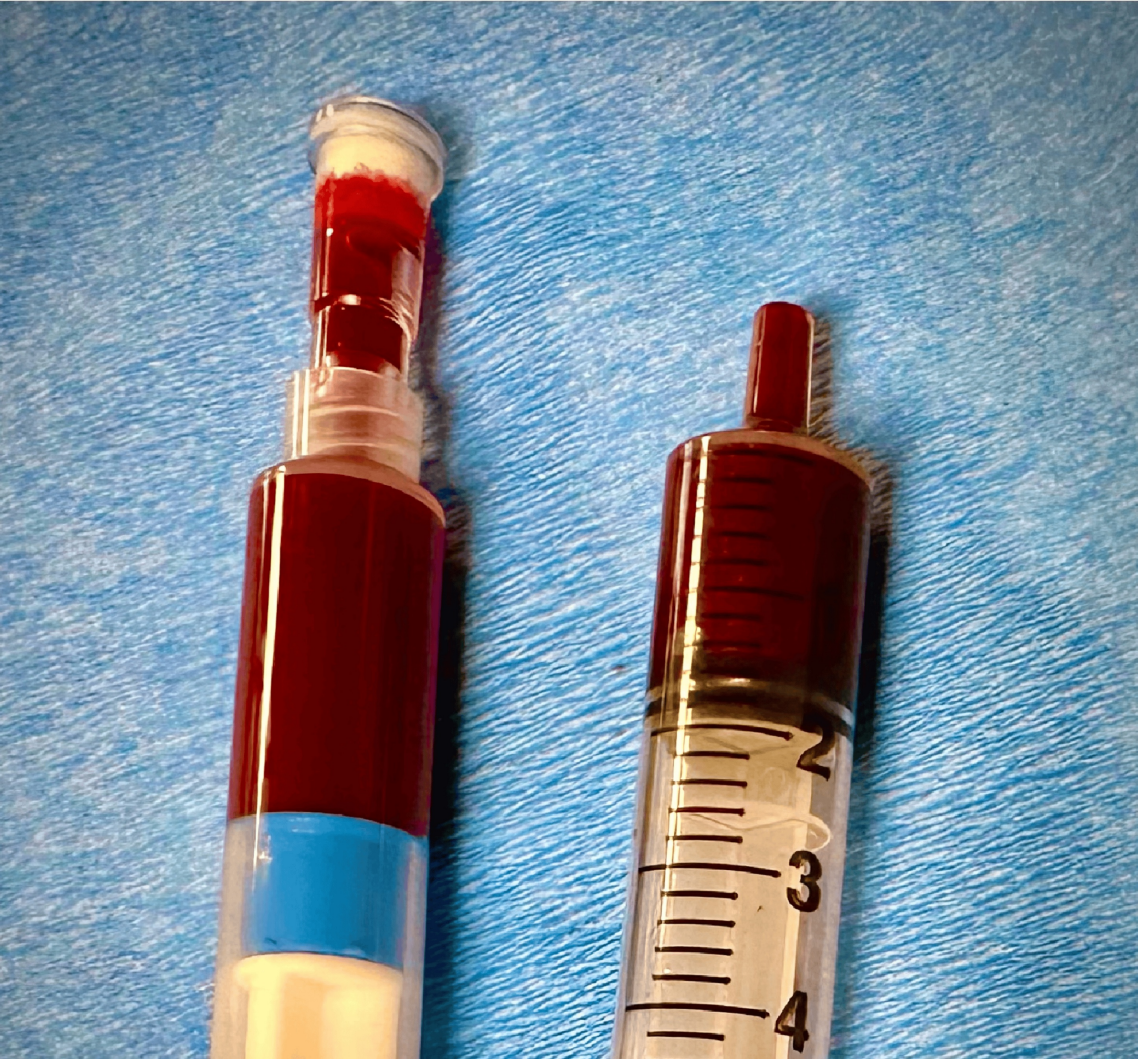
Difference in blood color between needle and catheter insertions Dark-colored venous blood seen on the right compared to bright blood color on the left.

**Figure 2:**
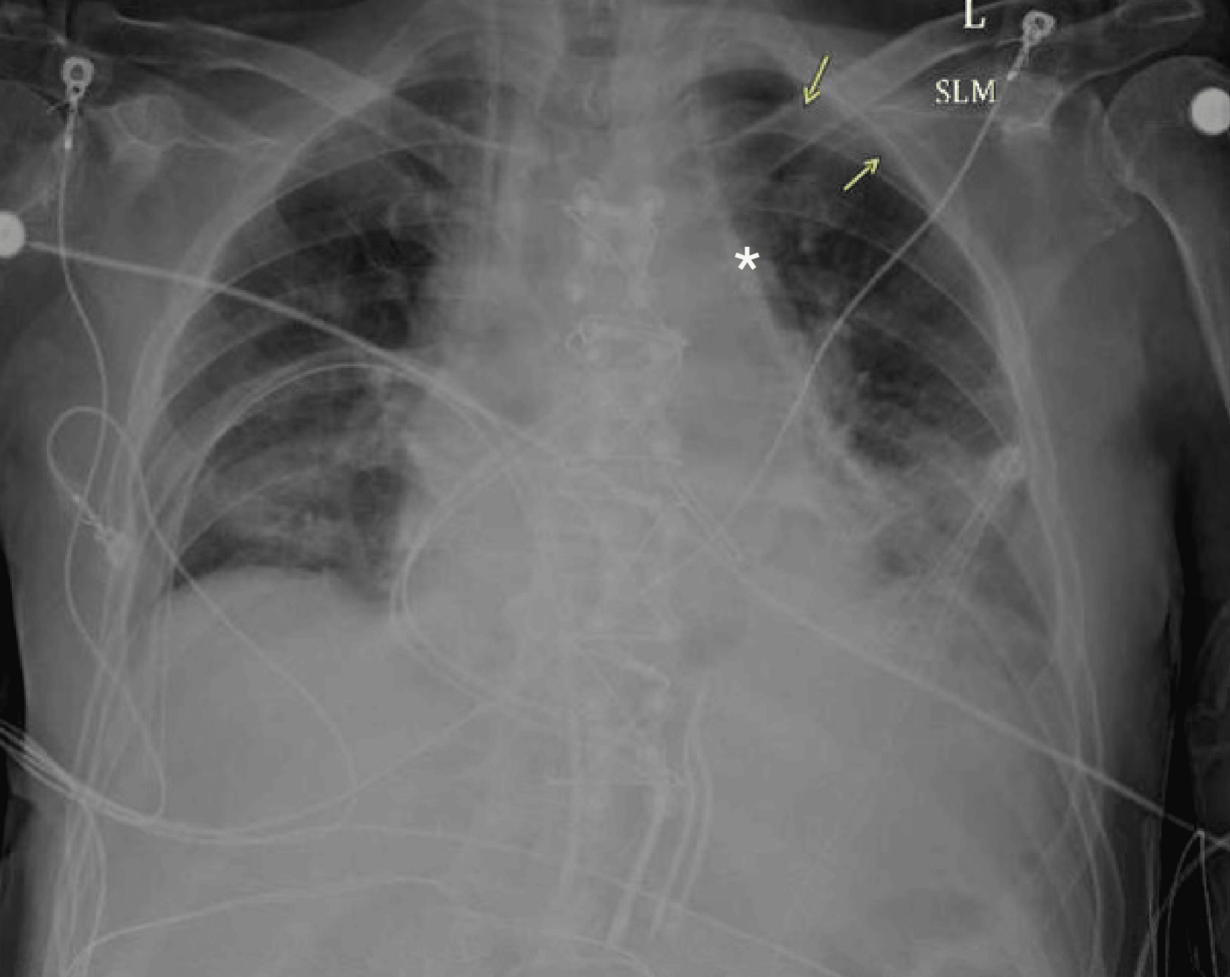
Plain chest x-ray showing malposition of left internal jugular central venous catheter placement Central venous catheter seen extending to left mediastinum (star). Small pneumothorax also present unchanged from before line placement (yellow arrows).

Nephrology was actively following the patient’s worsening renal function, and a decision was made to place a central line for potential CRRT, a continuous dialysis method used in critically ill patients to maintain hemodynamic stability. A non-tunneled dialysis catheter was attempted in the left internal jugular vein. The initial blood return from the needle was of venous color, and after confirmation of jugular guidewire placement with ultrasound, the dilator and dialysis catheter were inserted without difficulty. However, upon checking the catheter ports, the blood return appeared much brighter in color, raising concern for potential malpositioning (Figure 1). The catheter was transduced, showing pressures of 29/20, which were not suggestive of arterial placement. However, a plain chest x-ray revealed the catheter extending towards the left side of the mediastinum, raising suspicion of an anomalous vessel placement (Figure 2).

**Figure 3:**
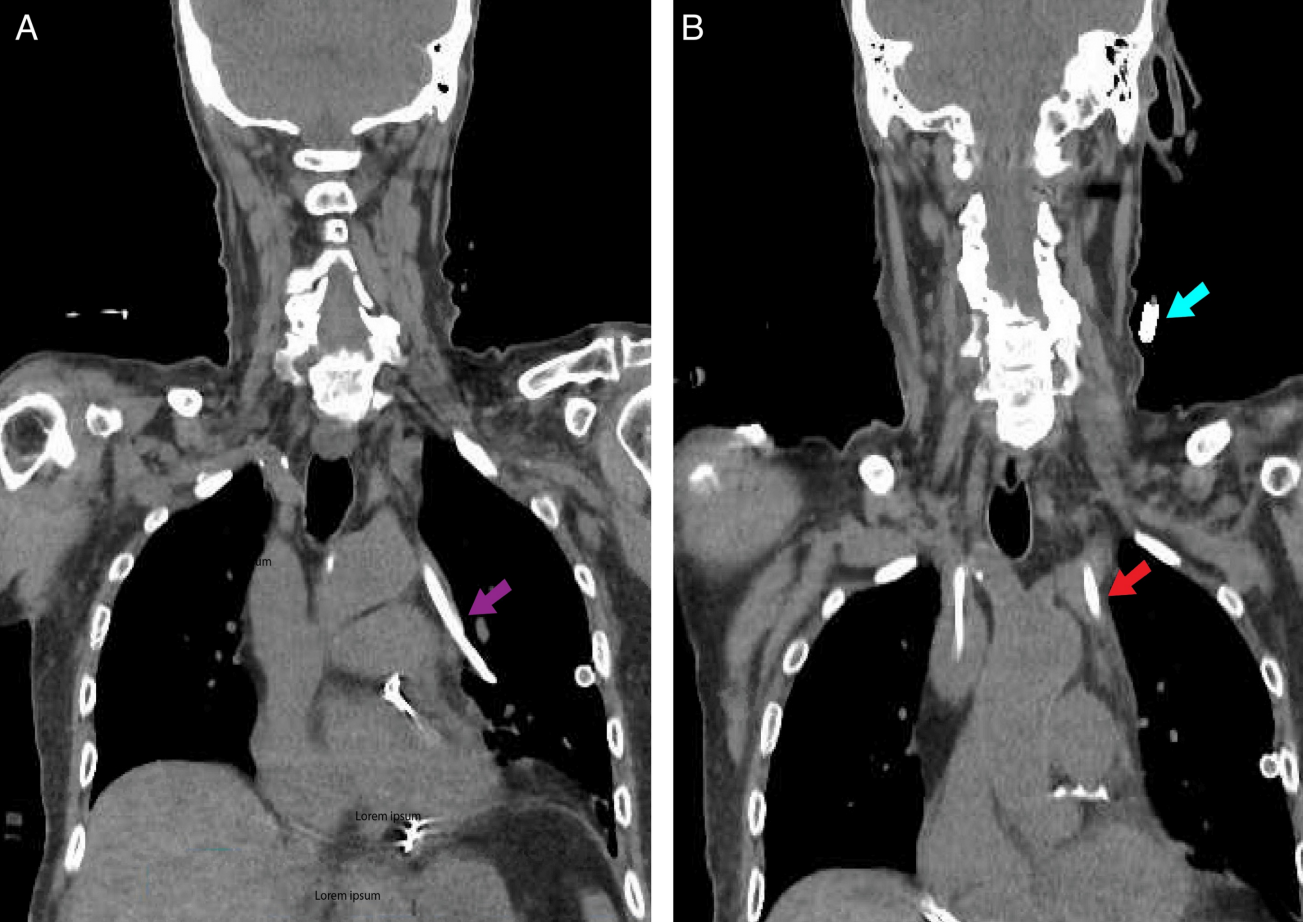
CT neck and chest without contrast showing central line visible in anomalous left upper lobe pulmonary vein CVC placed in partially anomalous pulmonary venous return (PAPVR) (A) CT imaging confirming the catheter was inadvertently placed in an anomalous left pulmonary vein (purple arrow). (B) The blue arrow showing proximal catheter entry point into a left internal jugular. The red arrow tracking the insertion of the pulmonary vein to the left innominate vein. CVC: Central venous catheter

Subsequent non-contrast CT imaging of the neck and chest (Figures 3A, 3B) confirmed that the dialysis catheter had been inadvertently placed in an anomalous left upper lobe pulmonary vein which appears to be draining in the left brachiocephalic vein. There was a tiny left apical pneumothorax seen on previous imaging with bilateral chest tubes that were in place previously.

Vascular surgery was consulted and recommended leaving the catheter in place and flushing it regularly to avoid further complications. Despite this rare complication, the patient remained hemodynamically stable, and there was no indication for immediate catheter removal. Over the following days, his respiratory and renal statuses improved, with weaning from inotropic support and high-flow oxygen. The patient was eventually discharged in stable condition to a skilled nursing facility for further rehabilitation, with follow-up planned for his multiple medical issues, including his pulmonary venous anomaly.

## Discussion

PAPVR, first described by Winslow in 1739, results from persistent embryonic venous anastomosis [7]. During normal embryological development, pulmonary veins originate as a common pulmonary venous plexus that drains into the splanchnic venous system before establishing a definitive connection with the left atrium. Around the fourth to sixth weeks of gestation, the common pulmonary vein forms as an outgrowth of the left atrium and eventually absorbs the pulmonary venous plexus, allowing for direct drainage into the left atrium. Failure of this process, due to incomplete incorporation of the pulmonary veins or persistent embryologic venous connections, can lead to PAPVR. This anomaly occurs when one or more, but not all, pulmonary veins retain abnormal connections to systemic veins instead of fully integrating into the left atrium [8].

The most common PAPVR defect occurs on the right side, typically draining into the superior vena cava (SVC) [2-5]. Less commonly, it involves the left pulmonary vein, often with the left upper pulmonary vein draining into the innominate vein, as seen in our case. The majority of cases are asymptomatic, and patients can escape diagnosis.

Misplacement of CVCs into PAPVR-associated pulmonary veins has been sporadically reported, with most cases discovered via imaging post-procedure [9,10]. As imaging modalities such as CT and MRI become more commonplace in clinical practice, it is expected that both the diagnosis of PAPVR and incidental CVC misplacements will increase [10]. Despite being a rare event, this finding underscores the importance of careful evaluation when venous access complications arise, particularly in patients with undiagnosed congenital anomalies. Management typically involves the removal and repositioning of the catheter, though the decision to treat the PAPVR itself remains nuanced. The management approach is based on factors such as RV function, pulmonary hypertension risk, and shunt volume, and those who are at low risk can be managed conservatively [11,12].

Functional impairment, right ventricular (RV) enlargement, and a high shunt fraction (Qp/Qs >1.5) are the primary indications for surgical intervention (Class 1B) [5,13-15]. Patients requiring surgery were often reported to have multiple anomalous pulmonary veins and RV enlargement, aligning with previous observations that cardiac decompensation is unlikely when less than half of pulmonary venous return is anomalous, and the presence of multiple anomalous veins or increased left atrial pressure can lead to preferential right atrial shunting [15]. While pulmonary hypertension is common, especially in older patients, Pulmonary artery systolic pressure should be less than 50% of systemic pressure and pulmonary vascular resistance less than 30% of one-third of systemic resistance to be considered for surgery (Class 1B) [14]. This aligns with recommendations for closing other left-to-right shunts, such as atrial septal defects, to prevent severe RV dysfunction post-repair. Fortunately, when present, preoperative RV dysfunction is typically mild, and surgical outcomes are generally excellent [16].

## Conclusions

This case underscores the importance of recognizing rare complications like CVC misplacement into an anomalous pulmonary vein and the need for exploring the approach to the incidental discovery of such anomalies. Identifying patients who may require further investigations or intervention.

## References

[1] Roldan CJ, Paniagua L (2015). Central venous catheter intravascular malpositioning: causes, prevention, diagnosis, and correction. West J Emerg Med.

[2] Haramati LB, Moche IE, Rivera VT (2003). Computed tomography of partial anomalous pulmonary venous connection in adults. J Comput Assist Tomogr.

[3] Ho ML, Bhalla S, Bierhals A, Gutierrez F (2009). MDCT of partial anomalous pulmonary venous return (PAPVR) in adults. J Thorac Imaging.

[4] Hatipoglu S, Almogheer B, Mahon C (2021). Clinical significance of partial anomalous pulmonary venous connections (isolated and atrial septal defect associated) determined by cardiovascular magnetic resonance. Circ Cardiovasc Imaging.

[5] El-Kersh K, Homsy E, Daniels CJ, Smith JS (2019). Partial anomalous pulmonary venous return: a case series with management approach. Respir Med Case Rep.

[6] Smith-Bindman R, Kwan ML, Marlow EC (2019). Trends in use of medical imaging in US health care systems and in Ontario, Canada, 2000-2016. JAMA.

[7] Ammash NM, Seward JB, Warnes CA, Connolly HM, O'Leary PW, Danielson GK (1997). Partial anomalous pulmonary venous connection: diagnosis by transesophageal echocardiography. J Am Coll Cardiol.

[8] Dillman JR, Yarram SG, Hernandez RJ (2009). Imaging of pulmonary venous developmental anomalies. AJR Am J Roentgenol.

[9] Chintu MR, Chinnappa S, Bhandari S (2008). Aberrant positioning of a central venous dialysis catheter to reveal a left-sided partial anomalous pulmonary venous connection. Vasc Health Risk Manag.

[10] Talukder S, Srinath SR, Behera A, Ganeshan D (2017). Accidental catheterization of left partial anomalous pulmonary venous connection (PAPVC): a rare instance of central venous access malpositioning. J Thorac Cardiovasc Surg.

[11] Hegde M, Manjunath SC, Usha MK (2019). Isolated partial anomalous pulmonary venous connection: development of volume overload and elevated estimated pulmonary pressure in adults. J Clin Imaging Sci.

[12] Edwin F (2010). Left-sided partial anomalous pulmonary venous connection--should diagnosis lead to surgery?. Interact Cardiovasc Thorac Surg.

[13] Ye R, Jiang Z (2022). Difficulty in central venous catheter placement due to congenital partial anomalous pulmonary venous return: a case report. Pulm Circ.

[14] Stout K, Daniels C, Aboulhosn J (2019). 2018 AHA/ACC guideline for the management of adults with congenital heart disease: a report of the American College of Cardiology/American Heart Association Task Force on clinical practice guidelines. J Am Coll Cardiol.

[15] Majdalany DS, Phillips SD, Dearani JA, Connolly HM, Warnes CA (2010). Isolated partial anomalous pulmonary venous connections in adults: twenty-year experience. Congenit Heart Dis.

[16] Sachweh JS, Daebritz SH, Hermanns B, Fausten B, Jockenhoevel S, Handt S, Messmer BJ (2006). Hypertensive pulmonary vascular disease in adults with secundum or sinus venosus atrial septal defect. Ann Thorac Surg.

